# Reversible suppression of circadian-driven locomotor rhythms in mice using a gradual fragmentation of the day-night cycle

**DOI:** 10.1038/s41598-023-41029-0

**Published:** 2023-09-02

**Authors:** Melissa E. S. Richardson, Chérie-Akilah Browne, Citlali I. Huerta Mazariegos

**Affiliations:** Department of Biological Sciences, Oakwood University, 7000 Adventist Blvd., Huntsville, AL 35896 USA

**Keywords:** Health care, Neuroscience, Circadian rhythms and sleep, Stress and resilience, Neurophysiology

## Abstract

Circadian rhythms are regulated by molecular clockwork and drive 24-h behaviors such as locomotor activity, which can be rendered non-functional through genetic knockouts of clock genes. Circadian rhythms are robust in constant darkness (DD) but are modulated to become exactly 24 h by the external day-night cycle. Whether ill-timed light and dark exposure can render circadian behaviors non-functional to the extent of genetic knockouts is less clear. In this study, we discovered an environmental approach that led to a reduction or lack in rhythmic 24-h-circadian wheel-running locomotor behavior in mice (referred to as arrhythmicity). We first observed behavioral circadian arrhythmicity when mice were gradually exposed to a previously published disruptive environment called the fragmented day-night cycle (FDN-G), while maintaining activity alignment with the four dispersed fragments of darkness. Remarkably, upon exposure to constant darkness (DD) or constant light (LL), FDN-G mice lost any resemblance to the FDN-G-only phenotype and instead, exhibited sporadic activity bursts. Circadian rhythms are maintained in control mice with sudden FDN exposure (FDN-S) and fully restored in FDN-G mice either spontaneously in DD or after 12 h:12 h light–dark exposure. This is the first study to generate a light–dark environment that induces reversible suppression of circadian locomotor rhythms in mice.

## Introduction

Circadian rhythms control the daily reoccurrence of vital biological processes. The alignment of circadian rhythms with the 24 h solar day is known as circadian photoentrainment and is important for the coordination of many biological processes^1–4^. Aberrant light and dark exposure disrupt the natural day-night cycle and lead to vast circadian disruptions in mood, sleep, activity, metabolism, memory, and development^5–9^. Circadian photoentrainment varies with the availability of light and dark but is flexible^10–12^. Using T-cycles where the total day-night cycle is longer or shorter than 24 h, a limit of 22–26 h has been determined to support entrainment in mice and hamsters^13, 14^. Furthermore, the presence of multiple periods of light and dark such as LDLD7:5:7:5 has been shown to produce two rhythmic periods of activity, the evidence of which has been observed through gene expression and in vivo studies in rodents^12, 15–18^. Additionally, the phase response curve (PRC) theory, which uses light exposure at specific times of the activity period (often referred to as subjective night) to shift the onset of the circadian period, has been manipulated to induce arrhythmic behavior in hamsters^19–21^. These are some of the many studies that support the theory that the circadian system is highly adaptable and flexible, within the known limits of entrainment.

Constant dark condition is the only known environment to reliably support an intrinsic free-running period (23.5 h in mice) and is indicative of a functional molecular clock^22–24^. In contrast, constant 24-h light exposure (LL) has been shown to induce period lengthening (~ 25 h in mice) in a light intensity-dependent fashion in what is known as the Aschoff rule^22, 22, 26, 27^. However, a lengthened circadian period has also been observed in environments where both light and dark existed, indicating that the molecular clock may categorically encode light–dark information to drive different kinds of circadian rhythms (i.e. entrained vs lengthened rhythms)^13, 28–32^. In addition to disrupted circadian photoentrainment, period lengthening is associated with many negative behavioral effects on development and mood^33–35^. Despite this knowledge of how light and dark affect the clock, many questions remain. At what point can a disruptive environment fully abolish behaviors regulated by the circadian clock? Furthermore, if a light/dark disruption was gradually introduced, would there be adaptation or loss of circadian-regulated behaviors?

In this study, we gradually fragment the 8-h night of a 16 h light:8 h dark cycle (previously reported in^36, 37^) (referred to as FDN-G in this study) and discovered unexpected behavioral adaptation and a loss of behavioral circadian rhythmicity in mice. Even more exciting was the finding that the behavioral circadian arrhythmicity was reversible upon environmental manipulation. This exciting reversible circadian arrhythmicity phenotype was confirmed by four major results: (1) gradual exposure to the FDN cycle (FDN-G) suppresses circadian rhythms compared to sudden exposure to the same FDN cycle, but is spontaneously reversed in constant darkness, which indicates there may be a temporary disruption to the circadian molecular clockwork, (2) exposure to constant light following FDN-G reveals complete disorganization, and thus arrhythmicity, of locomotor rhythms, (3) the disruptive impact of FDN-G is reversed upon exposure to a 24-day-night cycle and finally, (4) following exposure to a 24-h day-night cycle, the responses of the FDN-G mice to constant darkness, constant light, and FDN-S (sudden exposure to FDN) are fully restored, suggesting the environment has a stronger modulatory role on clock-driven behavior than previously reported. This study broadens our understanding of how the timing and exposure to light and dark impact underlying circadian rhythms of locomotion and produces the surprising discovery that a gradual change in the environment is more detrimental to the clock-driven locomotor behavior than a sudden change in the light–dark environment.

## Results

### Wheel-running behavior is broadly distributed across the 24-h day during gradual exposure to the fragmented day-night cycle (FDN-G) compared to sudden exposure (FDN-S)

**Figure 1:**
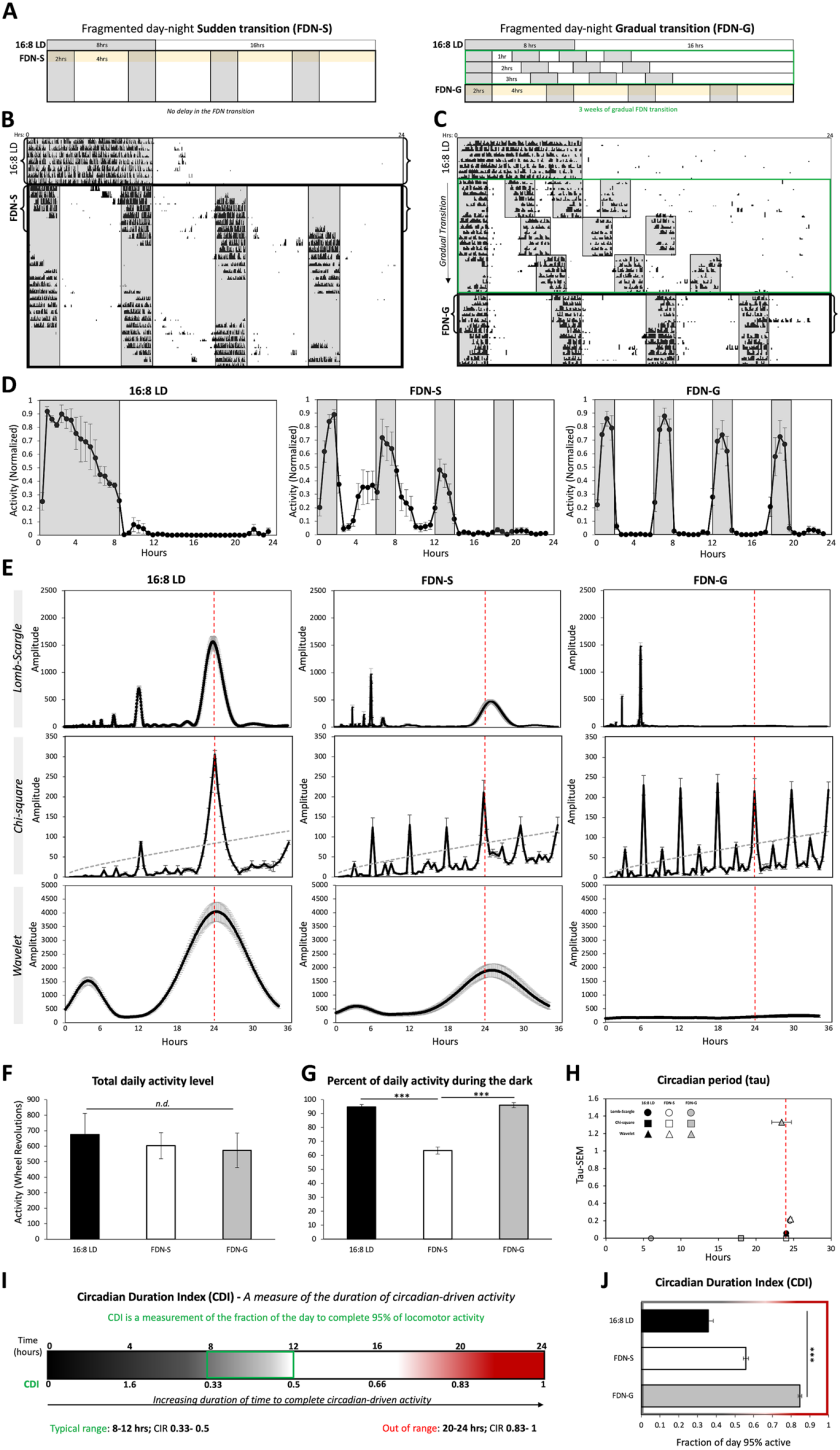
Wheel-running behavior is broadly distributed across the 24-h day during gradual exposure to the fragmented day-night cycle (FDN-G) compared to sudden exposure (FDN-S). (**A**) Schematic demonstrating the difference between the sudden exposure to the FDN cycle (FDN-S) and gradual exposure to the FDN cycle (FDN-G). Comparison between FDN-S and FDN-G occurred during the first 7 days as indicated by yellow shading. (**B**) Representative actograms of wheel-running activity during 16:8 LD to FDN-S (all FDN-S actograms used for analysis are in the supplementary Fig. S1. This actogram shows extra days for visual comparison with FDN-G over 4 weeks for the main figure only) and (**C**) 16:8 LD to FDN-G. *n* = 6 per group. Recording of wheel-running activity commenced as indicated on actograms (mice were in preceding LD cycles as explained in the “Methods” and Supplementary Tables S1). Brackets indicate periods of activity (7 days) used for further analysis. (**D**) Average of daily (across 7 days) wheel-running activity, collected in 30-min bins, and normalized to the highest daily activity for each mouse for 16:8 LD, FDN-S, and FDN-G. n = 6 per group. (**E**) Lomb-Scargle, Chi-square, and Wavelet periodograms averaged across 7 days for 16:8 LD, FDN-S, and FDN-G; the average (black line) is plotted with ± SEM error bars. Alignment with the 24-h day indicated by the dotted red line. *n* = 6 per group, comparison between all groups for Lomb-Scargle: *F* = 153.3, *P* < 0.0001***, peak average amplitudes: 1565.57, 473.99, 18.56, Range: 702.93, 189.75, 30.9, and ± SEM: 109.6, 29.5, 6.02 Chi-square: the dashed gray line indicated the significance line (0.05). *F* = 11.81, *P* < 0.001***, peak average amplitudes: 305.5, 211.31, 216.0, Range: 75.76, 125.79, 11.94, and ± SEM: 10.4, 17.88, 16.9. *Analysis of the daily peaks (Hours 6, 12, 18, 24) for FDN-S and FDN-G.* FDN-S: F = 9.58, P < 0.001***, peak average amplitudes: 123.5, 130, 124, 210.7, Range: 113.3, 110.9, 100.9, 125.8, and ± SEM: 12.8, 12.1, 12.6, 15.1. FDN-G: F = 0.19, P < 0.9, peak average amplitudes: 230, 223, 235, 215, Range: 140, 131, 131, 110, and ± SEM: 20, 19.4, 19.4, 17. Wavelet: *F* = 58.34, *P* < 0.0001***, peak average amplitudes: 4036.77, 1925.75, 219.59, Range: 2242.45, 1561.01, 280.28, and ± SEM: 350.47, 251.65, 43.14. (**F**) Average total wheel-running activity across 7 days for 16:8 LD, FDN-S, and FDN-G. *n* = 6 per group, *F* = 0.3161, *P* = 0.7337, ranges: 850.9, 430.4, and 688.3, and ± SEM: 136.67, 83.69, 110.93, *n.d.* = no significant difference. (**G**) Percent of total daily activity that occurred during the dark portion(s) for 16:8 LD, FDN-S, and FDN-G. *n* = 6 per group, *F* = 53.57,* P* < 0.0001***, ranges: 10.20, 28.4, 11.29, and ± SEM: 1.83, 2.57, 1.87. (**H**) Circadian period (tau). The average tau at the peak amplitude of periodograms in “E” is graphed (x-axis) vs. the SEM of the tau for each experimental group (y-axis). The ± SEM error bars for the average tau are also displayed. Alignment with the 24-h day indicated by the dotted red line. *n* = 6 per group, Lomb-Scargle: *F* = 15,583.98, *P* < 0.0001***, peak average tau: 23.95, 25.16, 6, Range: 0.45, 0.95, 0, and ± SEM: 0.06, 0.13, 0. Chi-square: *F* = 65,535, peak average tau: 24, 24, 18, Range: 0, 0, 0, and ± SEM: 0, 0, 0. Wavelet:* F* = 0.08, *P* < 0.922, peak average tau: 24.03, 24.61, 23.46, Range: 0.49, 2.33, 22.16, and ± SEM: 0.07, 0.33, 3.44. (**I**) Schematic of the Circadian Duration Index (CDI), demonstrating the relationship between the typical duration of mouse wheel-running locomotion and values that would be considered non-circadian and atypical. (**J**) Assessment of the period of circadian-driven wheel-running activity using the CDI for 16:8 LD, FDN-S, and FDN-G. *n* = 6 per group, *F* = 0.40, *P* < 0.0001***, and ranges: 3.50, 2.50, 2.50 ± SEM: 0.03, 0.01, 0.01.

Previous studies demonstrated that fragmenting the day-night cycle disrupted circadian photoentrainment, induced a lengthened period, increased activity during the light portion of the FDN cycle, and disrupted mood and development^36, 37^. Based on the previous findings, we hypothesized that there was a threshold for fragmenting the day-night cycle when mice would no longer exhibit circadian photoentrainment and switch to a lengthened circadian period (> 25 h). To determine the minimum level of fragmentation needed to disrupt circadian photoentrainment and induce period lengthening, the 4, 2-h fragments of darkness in the FDN cycle were gradually separated by 1 h each week, for a total transition time of 3 weeks, until the traditional 4-h light separation was accomplished for the FDN cycle, referred to as FDN-G (Fig. 1A and Supplementary Table S1). The first 7 days of the FDN-S and FDN-G experimental conditions were used for the analyses. The 7 days in 16:8 LD before FDN-S exposure was used as the control for the consolidated 8 h of darkness. Circadian distribution of locomotor activity across the day was maintained in FDN-S exposed mice (Fig. 1B,D) but absent in FDN-G exposed mice (Fig. 1C,D). There was no difference between the total daily activity level in 16:8 LD, FDN-S, and FDN-G conditions (Fig. 1F). FDN-G mice avoided light and restricted their activity to the dark fragments better than FDN-S mice similar to 16:8 LD mice (Fig. 1D,G).

The presence of a behavioral circadian rhythm, or periodicity, can be measured using multiple tools on the commonly used ClockLab software such as Lomb-Scargle periodogram, chi-square periodogram, and wavelet analysis, although there are contrasting views on their usage, based on the number of days being analyzed or the need to use multiple means of rhythmic analysis^38–40^. If a circadian rhythm is present, these tools will give both a numerical value (tau) (for example ~ 24-h or not) and a measure of confidence in the reported periodicity value (the amplitude). Therefore, in this study, low amplitude, a non-24-h circadian period, and a highly variable peak amplitude (of tau) measurement (determined by SEM of the peak amplitude of tau (between the mice within each experimental group): referred to as Tau-SEM henceforth) are defining factors of a weak or absent circadian rhythm (circadian arrhythmicity) of locomotor behavior. Compared to the period lengthening observed following FDN-S (Fig. 1B), mice gradually exposed to the FDN cycle (FDN-G) did not exhibit a distinguishable circadian period as analyzed by Lomb-Scargle periodogram and wavelet analysis (Fig. 1C,E, and Supplementary Fig. S1). The chi-square analysis of FDN-S and FDN-G exposed mice revealed noticeable peaks every 6 h that mirror the 4 h-light:2 h-dark cycles embedded within the FDN cycle (Fig. 1E). While the 24-h peak was statistically distinguishable from the non-24-h peaks for FDN-S mice (*F* = 9.58, *P* < 0.001), the 24-h peak for FDN-G mice was statistically indistinguishable from the other 6-h peaks (*F* = 0.19, *P* < 0.9), leading to our conclusion that the chi-square periodogram also displays an indistinguishably circadian pattern. FDN-G mice exhibited an equal and fragmented distribution of activity that aligned with the 4 dark periods in the FDN-G paradigm (Fig. 1C,D, and Supplementary Fig. S2). We found high variability in the range of tau values reported by the Lomb-Scargle periodogram, chi-square periodogram, and wavelet analysis for FDN-G compared to 16:8 LD and FDN-S (Fig. 1H).

Tools that measure periodicity do not capture the total time required to complete the circadian-driven locomotor behavior. To further understand how activity distribution changes in various light–dark environments, we designed a tool called Circadian Duration Index (CDI) that measures the fraction of the 24-h day needed to complete 95% of the total activity on a 0–1 scale. The typical range for circadian locomotion in our mice is 0.33–0.5 (33–50% of the day) (Fig. 1I). FDN-G mice exhibited wheel-running activity that was distributed across 20.33 h ± 0.36 (SEM), a high CDI value of 0.85 ± 0.01 (SEM), compared to the lower CDI values for 16:8 LD (0.36 ± 0.03 (SEM)) and FDN-S (0.56 ± 0.01 (SEM)) (Fig. 1J). Together, these findings indicate that gradual exposure to the FDN cycle results in improved acute light suppression of locomotor activity and a non-circadian or highly variable circadian distribution of locomotor activity.

### Intrinsic behavioral circadian rhythmicity is initially suppressed during constant dark exposure following FDN-G

**Figure 2:**
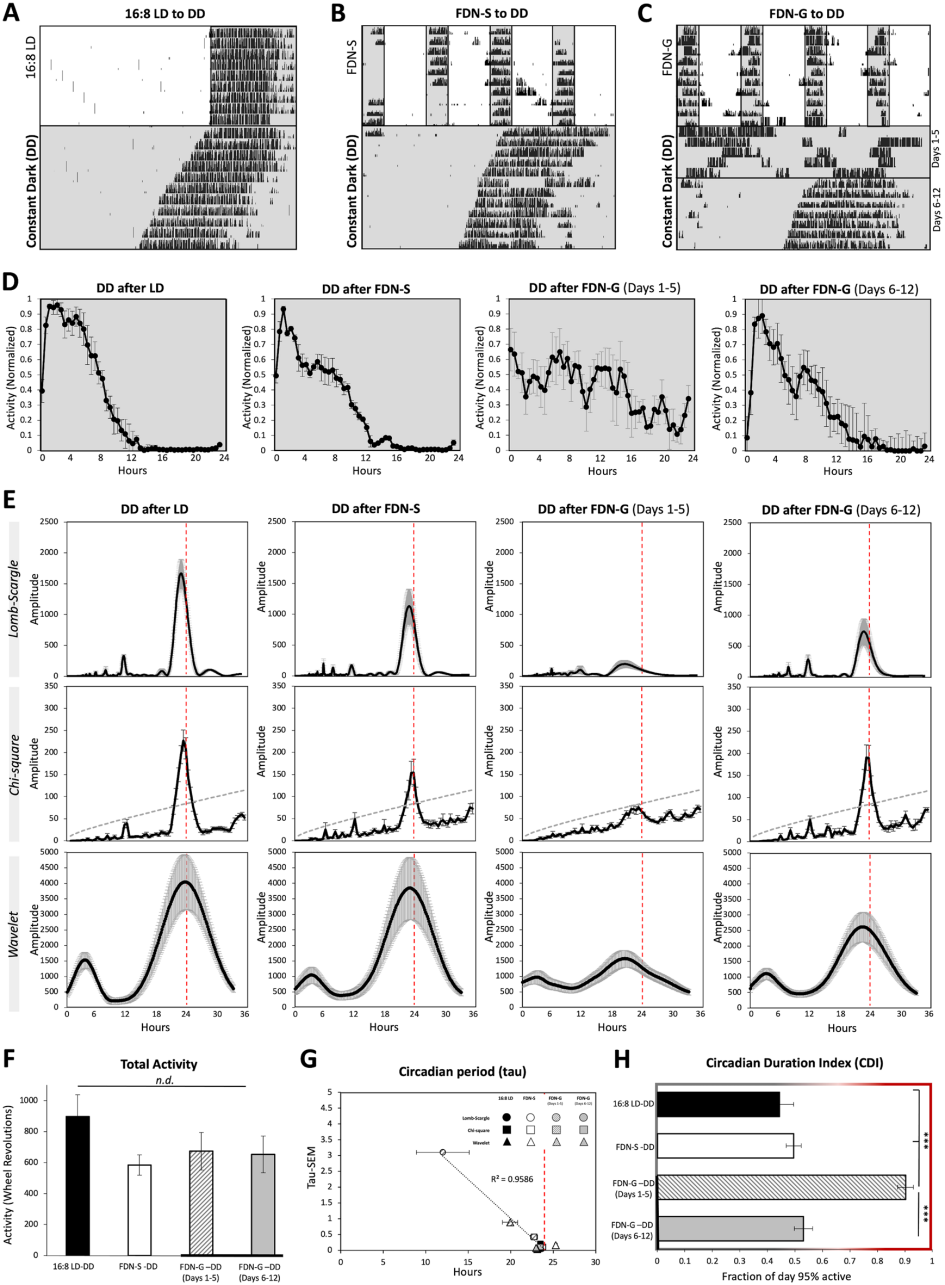
Intrinsic behavioral circadian rhythm is initially suppressed during constant dark exposure following FDN-G. Representative actograms of wheel-running activity during constant darkness following (**A**) 16:8 LD, (**B**) FDN-S, and (**C**) FDN-G. *n* = 6 per group. Recording of wheel-running activity commenced as indicated on actograms (mice were in preceding LD cycles as explained in the “Methods” and Supplementary Tables S1). The analysis for figures (**A**) and (**B**) are based on the first 7 days in DD. (**D**) Average of daily (across 7 days for all except FDN-G days 1–5) wheel-running activity, collected in 30-min bins, and normalized to the highest daily activity for each mouse. (**E**) Lomb-Scargle, Chi-square, and Wavelet periodograms averaged across 7 days for DD following 16:8 LD, FDN-S, and FDN-G; the average (black line) is plotted with ± SEM error bars. Alignment with the 24-h day indicated by the dotted red line. n = 6 per group, comparison between all groups for Lomb-Scargle: F = 9.52, P < 0.0001***, peak average amplitudes: 1671.82, 1132.36, 212.72, 740.94, Range: 1390.03, 1525.79, 399.08, 1369.8 and ± SEM: 228.28, 248.87, 57.56, 206.14. Chi-square: The dashed gray line indicates the significance line (0.05). *F* = 7.21, *P* < 0.002**, peak average amplitudes: 227, 153.8, 84.6, 189.1, range: 141.7, 182.88, 68.13, 149.33, and ± SEM: 24, 26, 10.6, 25.71. Wavelet: *F* = 2.88, *P* < 0.06, peak average amplitudes: 4542.83, 3338.5, 1924.5, 2702.67, Range: 5655, 4147, 2066, 3369 and ± SEM: 946.82, 670.24, 357.41, 473.52. (**F**) Average total wheel-running activity across 7 days. *n* = 6 per group, *F* = 1.33, *P* = 0.29, ranges: 867.4, 583.5, 739.9, 715.4 and ± SEM: 141.9, 76.94, 121.9, 118.4. (**G**) Circadian period (tau). The average tau at the peak amplitude of periodograms in “E” is graphed (x-axis) vs. the SEM of the tau for each experimental group (y-axis). The ± SEM error bars for the average tau are also displayed. Alignment with the 24-h day indicated by the dotted red line. *n* = 6 per group, Lomb-Scargle: *F* = 8.26, *P* < 0.001***, peak average tau: 23.56, 23.62, 14.29, 23.35, Range: 0.25, 0.25, 16.35, 0.55, and ± SEM: 0.04, 0.05, 3.1, 0.07. Chi-square: *F* = 3.1, *P* < 0.05*, peak average tau: 23.42, 23.66, 22.75, 23.66, Range: 1.5, 0.5, 3, 0.5, and ± SEM: 0.2, 0.1, 0.42, 0.1. Wavelet:* F* = 0.98, *P* < 0.42, peak average tau: 23.13, 23.69, 19.92, 23, Range: 0.5, 2.51, 21.14, 1.83, and ± SEM: 0.07, 0.39, 3.39, 0.31. (**H**) Assessment of the period of circadian-driven wheel-running activity using the Circadian Duration Index (CDI). *n* = 6 per group, *F* = 48.65, *P* < 0.0001***, and ranges: 4.50, 5.00, 5.00, 5.00 ± SEM: 0.66, 0.76, 0.69, 0.79.

Analysis of the intrinsic circadian rhythm, which occurs in the absence of light stimuli, informs us of the internal rhythmic state of the clock, which typically exhibits a strong circadian period of approximately 23.5 h in mice (Refs.^22–24^ and Fig. 2A,D,E, and Supplementary Fig. S3). The first 7 days of experimental conditions were used for analysis except for FDN-G to DD, which were split into 5 days and 7 days. To determine if the FDN-S and FDN-G cycles were detrimental to the ability of the intrinsic clock to drive locomotion, we removed light stimuli following FDN-S and FDN-G and observed the activity rhythms (Fig. 2B,C, and Supplementary Table S2). The level of wheel-running activity was similar in DD for all experimental groups (Fig. 2F). During DD but following FDN-S and 16:8 LD, mice immediately switched to a shortened circadian period and exhibited strong amplitude peaks on the Lomb-Scargle, chi-square, and wavelet periodograms (Fig. 2A,B,E,G). However, when exposed to DD for 12 days, FDN-G mice exhibit an initial suppression of rhythmicity (peak amplitude):* F* = 9.52, P < 0.0001 (Lomb-Scargle), *F* = 7.21, *P* < 0.002 (Chi-square), and *F* = 2.88, *P* < 0.06 (Wavelet) with variable circadian periodicity detection (tau): *F* = 8.26, *P* < 0.001 (Lomb-Scargle), *F* = 3.1, *P* < 0.05 (Chi-square), and *F* = 0.98, *P* < 0.42 (Wavelet) (Fig. 2E,G) and a high CDI score of 0.90 ± 0.69 (SEM) (*F* = 48.65, *P* < 0.0001) during the first 5 days in DD (Fig. 2H). However, during the latter portion of the DD exposure period (days 6–12), the previously exposed FDN-G mice recovered a normal shortened circadian rhythm of 23.65 h ± 0.04 (SEM), increased amplitude similar to FDN-S and 16:8 LD mice and exhibited a low CDI score of 0.53 ± 0.79 (SEM) (Fig. 2C–E,G,H). Together, these findings confirm that gradual exposure to the FDN cycle results in temporary loss of a strong locomotor circadian rhythm in DD, which lasted for the first 5 days of the total 12 days in DD.

### Loss of behavioral circadian rhythmicity occurs during exposure to constant light (LL) following FDN-G

**Figure 3:**
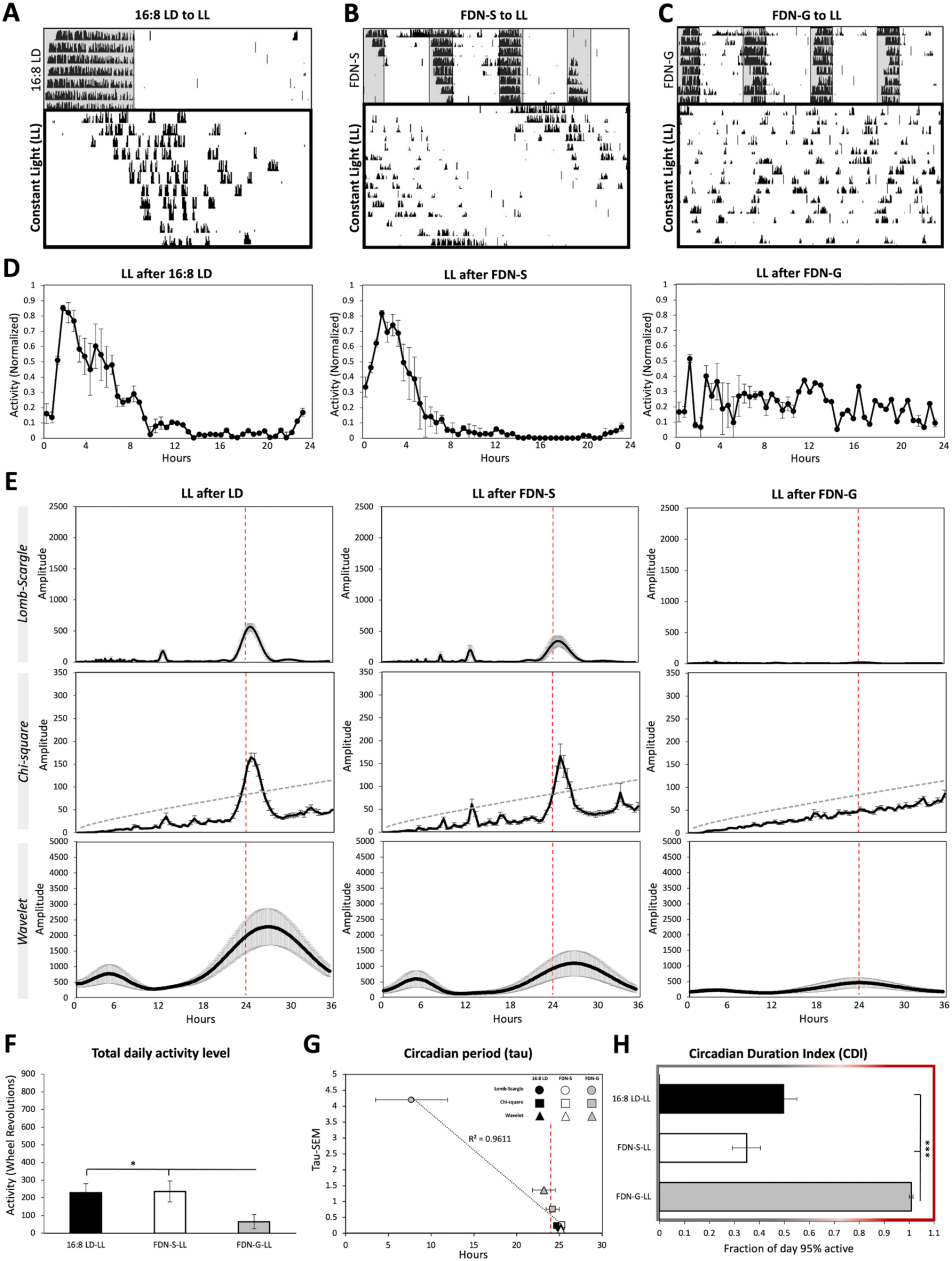
Loss of behavioral circadian rhythm occurs during exposure to constant light (LL) following FDN-G. Representative actograms of wheel-running activity during constant light following (**A**) 16:8 LD, (**B**) FDN-S, and (**C**) FDN-G. *n* = 6 per group. Recording of wheel-running activity commenced as indicated on actograms (mice were in preceding LD cycles as explained in the “Methods” and Supplementary Tables S1). The analysis is based on the first 7 days in LL. (**D**) Average of daily (across 7 days) wheel-running activity, collected in 30-min bins, and normalized to the highest daily activity for each mouse. (**E**) Lomb-Scargle, Chi-square, and Wavelet periodograms averaged across 7 days for LL following 16:8 LD, FDN-S, and FDN-G; the average (black line) is plotted with ± SEM error bars. Alignment with the 24-h day indicated by the dotted red line. n = 6 per group, comparison between all groups for Lomb-Scargle: F = 29.04, P < 0.0001***, peak average amplitudes: 578.47, 354.71, 17, Range: 362.95, 586.7, 31, and ± SEM: 56.91, 91.05, 4.21. Chi-square: The dashed gray line indicates the significance line (0.05). *F* = 30.37, *P* < 0.0001***, peak average amplitudes: 172.2, 171.5, 60.28, Range: 60.8, 114, 20, and ± SEM: 10.5, 17, 3. Wavelet: *F* = 4.86, *P* < 0.02*, peak average amplitudes: 2313.5, 1091.833, 488, Range: 3460, 2722, 1073 and ± SEM: 581.77, 405.99, 171.94. (**F**) Average total wheel-running activity across 7 days. *n* = 6 per group, *F* = 4.16, *P* = 0.036*, ranges: 308.0, 341.1, 230.1, and ± SEM: 42.86, 59.45, 35.02. (**G**) Circadian period (tau). The average tau at the peak amplitude of periodograms in “E” is graphed (x-axis) vs. the SEM of the tau for each experimental group (y-axis). The ± SEM error bars for the average tau are also displayed. Alignment with the 24-h day indicated by the dotted red line. *n* = 6 per group, Lomb-Scargle: *F* = 9.31, *P* < 0.002**, peak average tau: 24.8, 25.06, 11.04, Range: 1, 1, 23.8, and ± SEM: 0.14, 0.17, 4.54. Chi-square: *F* = 1.86, *P* < 0.18, peak average tau: 24.66, 25.4, 26, Range: 1.5, 1.5, 5, and ± SEM: 0.25, 0.27, 0.76. Wavelet:* F* = 3.8, *P* < 0.046*, peak average tau: 25.19, 25.19, 23.18, Range: 3.66, 1.16, 5.16, and ± SEM: 0.52, 0.18, 0.86. (**H**) Assessment of the period of circadian-driven wheel-running activity using the Circadian Duration Index (CDI). *n* = 6 per group, *F* = 43.75, *P* < 0.0001***, and ranges: 11.00, 9.00, 1.00. ± SEM: 1.49, 1.37, 0.20.

In addition to FDN-S, period lengthening is strongly associated with constant light conditions, which is considered highly disruptive to circadian photoentrainment and circadian-driven behaviors (Refs.^22, 33, 35^, Fig. 3A, and Supplementary Fig. S4). The first 7 days of experimental conditions were used for analysis. We hypothesized that since circadian period lengthening is observed in FDN-S, FDN-S mice would continue to lengthen their circadian period when exposed to LL, considering that previous studies showed that LL and FDN-S had similar behavioral phenotypes^36^. We also hypothesized that if FDN-G mice maintain any circadian rhythms, LL would induce period lengthening in FDN-G-exposed mice. Mice exposed to LL following FDN-S exhibited a continuous and extended period lengthening rhythm from FDN-S to LL, with a circadian period similar to mice previously exposed to 16:8 LD (Fig. 3B,D,E,G, and Supplementary Table S3). In contrast to the period-lengthening phenotype in FDN-S, mice exposed to LL following FDN-G lost their circadian rhythm, exhibiting no distinguishable peak on any of the three periodicity tools, and exhibiting significant differences in the peak amplitude:* F* = 29.04, P < 0.0001 (Lomb-Scargle), *F* = 30.37, *P* < 0.0001 (Chi-square), and *F* = 4.86, *P* < 0.02 (Wavelet) with variable circadian periodicity detection (tau): *F* = 9.31, *P* < 0.002 (Lomb-Scargle), *F* = 1.86, *P* < 0.18 (Chi-square), and *F* = 3.8, *P* < 0.046 (Wavelet) (Fig. 3E,G). There was a reduction in wheel-running activity during LL following 16:8 LD, FDN-S, and FDN-G conditions, with previously FDN-G-exposed mice exhibiting the largest reduction (*P* = 0.036) in activity (Fig. 3F). Low CDI scores represent the daily consolidation of circadian-driven locomotion observed in LL following 16:8 LD and FDN-S (Fig. 3D,H). Circadian rhythms were abolished in LL for FDN-G exposed mice which exhibited a high CDI score of 1.00 ± 0.01 (SEM), which indicates that most of the locomotor activity was distributed across 24 h instead of being restricted to 12 h and 8.5 h in LL-exposed 16:8 LD and FDN-S mice, respectively (Fig. 3D,H). These findings indicate that gradual exposure to the FDN environment leads to loss of circadian rhythmicity during constant light where a circadian rhythm of ~ 25 h is usually maintained.

### The effects of the FDN-G on locomotion are temporary: normal rhythms are recoverable with 1 week of LD exposure

**Figure 4:**
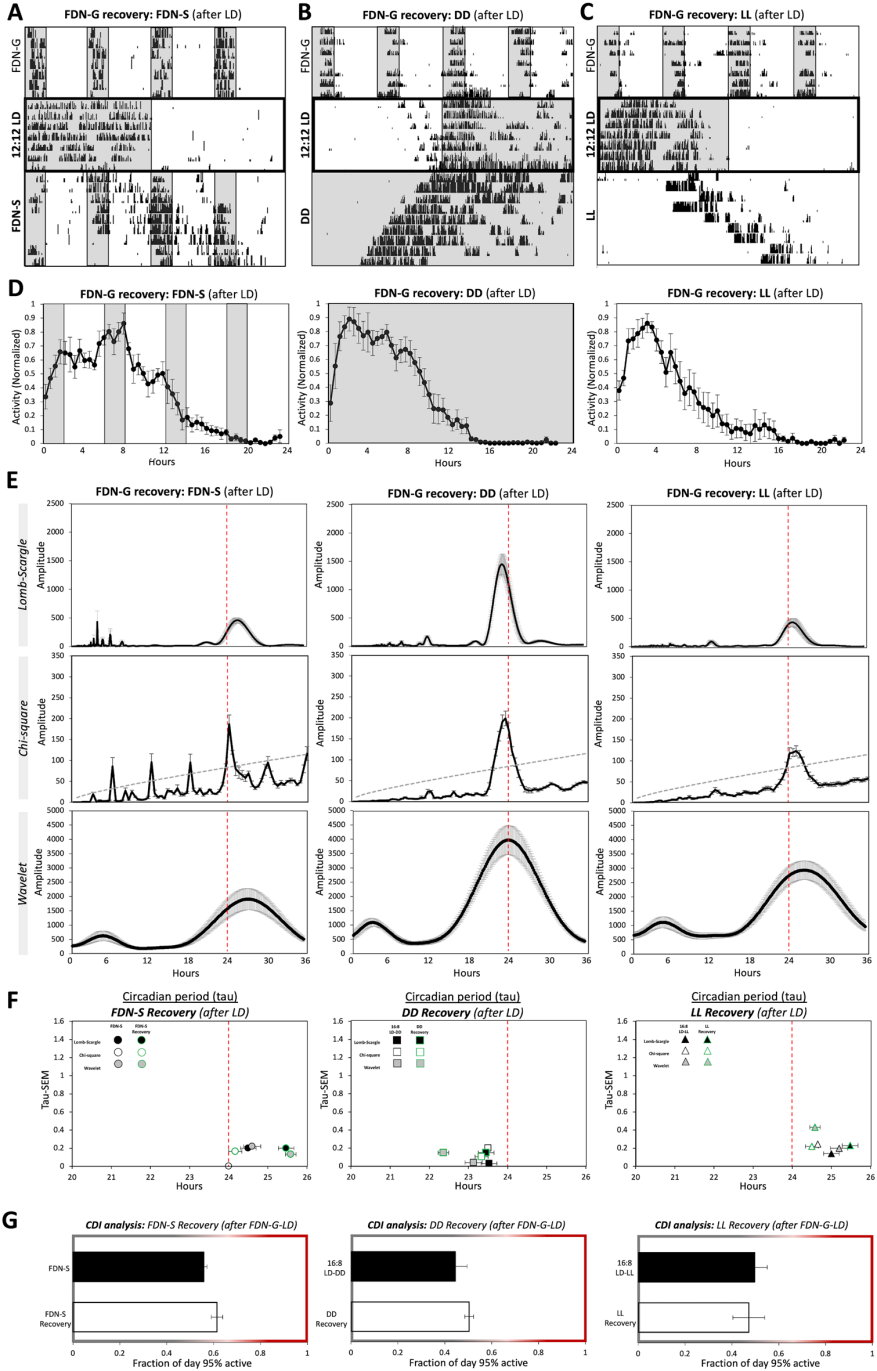
The effects of the FDN-G on locomotion are temporary. Normal rhythms are recoverable with 1 week of LD exposure. (**A**) Representative actograms of wheel-running activity during (**A**) FDN-S, (**B**) DD, and (**C**) LL following FDN-G and 12:12 LD exposure. *n* = 6 per group. Recording of wheel-running activity commenced as indicated on actograms (mice were in preceding LD cycles as explained in the “Methods” and Supplementary Tables S1). The analysis is based on the first 7 days in FDN-S, DD, and LL. (**D**) Average of daily (across 7 days) wheel-running activity, collected in 30-min bins, and normalized to the highest daily activity for each mouse. (**E**) Lomb-Scargle, Chi-square, and Wavelet periodograms averaged across 7 days for FDN-S, DD, and LL following FDN-G and 12:12 LD exposure; the average (black line) is plotted with ± SEM error bars. Alignment with the 24-h day indicated by the dotted red line. n = 6 per group, comparison between all recovery groups for Lomb-Scargle: F = 25.5, P < 0.0001***, peak average for circadian amplitudes: 477.54, 1506.7, 454.51, Range: 269.73, 1297.49, 574.47 and ± SEM: 43.9, 185.62, 78.06. Chi-square: The dashed gray line indicates the significance line (0.05). *F* = 5.47, *P* < 0.01**, peak average amplitudes: 186.9, 206.59, 128.6, Range: 134.5, 123.5, 76.8, and ± SEM: 21.8, 17.9, 10.3. Wavelet: *F* = 6.87, *P* < 0.007**, peak average amplitudes: 1919, 4021.33, 3057.167, Range: 2118, 2944, 2318, and ± SEM: 373.03, 494.33, 316.04. (**F**) Circadian period (tau). The average tau at the peak amplitude of periodograms in “E” is graphed (x-axis) vs. the SEM of the tau for each experimental group (y-axis). The ± SEM error bars for the average tau are also displayed. Previous data included for comparison: FDN-S (Fig. 1), 16:8LD-DD (Fig. 2), and 16:8 LD-LL (Fig. 3). Alignment with the 24-h day indicated by the dotted red line. *n* = 6 per group, Lomb-Scargle: *F* = 37.77, *P* < 0.0001***, peak average tau: 25.76, 23.33, 24.96, Range: 1.3, 1, 1.4, and ± SEM: 0.21, 0.15, 0.23. Chi-square: *F* = 12.19, *P* < 0.001***, peak average tau: 24.16, 23.33, 24.5, Range: 1, 0.5, 1, and ± SEM: 0.16, 0.1, 0.22. Wavelet:* F* = 11.45, *P* < 0.001***, peak average tau: 25.58, 22.36, 24.58, Range: 1.83, 2, 4.67, and ± SEM: 0.28, 0.31, 0.72. G) Assessment of the period of circadian-driven wheel-running activity using the Circadian Duration Index (CDI) during FDN-S (*n* = 6, *F* = 2.67, *P* = 0.18, range: 2.50, 3.50, and ± SEM: 0.35, 0.58), DD (*n* = 6, *F* = 2.13, *P* = 0.07, range: 4.50, 2.50, and ± SEM: 0.66, 0.45), and LL (*n* = 6, *F* = 1.24, *P* = 0.79, range: 11.00, 10.50 and ± SEM: 1.47, 1.64), compared to mice previously exposed to 16:8 LD only.

In a previous study, we showed that re-introduction to a 12:12 LD cycle following FDN-S reversed negative effects on mood in 1–2 weeks^36^. Therefore, we wanted to determine if exposure to 12:12 LD could recover circadian photoentrainment as well as the shortened and lengthened circadian rhythms associated with DD, LL, and FDN-S, respectively, in FDN-G-exposed mice. FDN-G mice exposed to 1-week of 12:12 LD showed immediate recovery of an entrained 24-h circadian rhythm (Fig. 4A–C—middle panel, and Supplementary Fig. S5). The first 7 days of experimental conditions were used for analysis. When previously FDN-G-exposed mice were suddenly re-exposed to the FDN cycle (FDN-S) following 12:12 LD, they demonstrated recovery of a circadian rhythm, increased amplitude, and exhibited the period-lengthening phenotype previously observed with the FDN-S mice (Fig. 4A,D–F, and Supplementary Table S4). Similarly, exposure of FDN-G mice to DD and LL after 1 week in 12:12 LD yields a recovery of circadian rhythms, increased amplitudes, and circadian periods typical of DD and LL following an LD cycle (Fig. 4B–F and Supplementary Fig. S5). The recovered activity distribution (CDI analysis) of the FDN-G mice was not different from the control mice that were only exposed to an LD cycle before FDN-S, DD, or LL (Fig. 4G). These findings indicate that the disrupted circadian rhythm during FDN-G is reversible with 1 week of 12:12 LD exposure.

### Highly variable circadian periods are associated with a broad distribution of activity across the day

**Figure 5:**
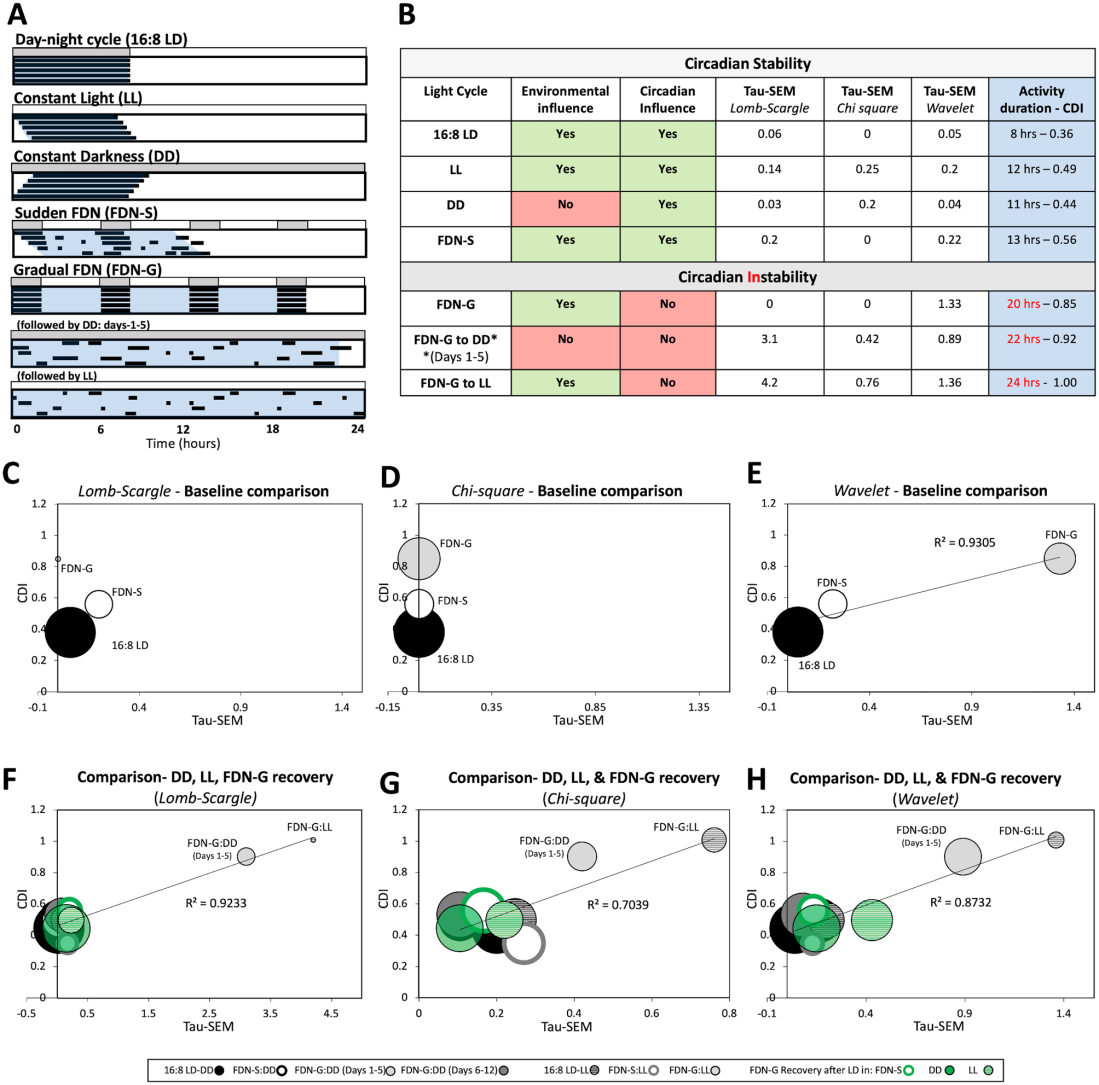
Highly variable circadian periods are associated with a broad distribution of activity across the day. (**A**) Schematic of key light environments from our study (activity duration in blue shading) within the context of the 24-h day are highlighted. (**B**) A comparison of light environment, detectable circadian rhythms (within the control-like range), and behavioral outputs. Tau-SEM is the variability measure of the tau measured at the peak amplitude using the Lomb-Scargle, Chi-square, and Wavelet periodograms. (**C**) Comparison of baseline CDI (y-axis) with Tau-SEM (x-axis) and amplitude (bubble size) from the Lomb-Scargle analysis for data from Fig. 1. (**D**) Comparison of baseline CDI (y-axis) with Tau-SEM (x-axis) and amplitude (bubble size) from the Chi-square analysis for data from Fig. 1. (**E**) Comparison of baseline CDI with Tau-SEM from the Wavelet analysis for data from Fig. 1. (**F**) Comparison of baseline CDI (y-axis) with Tau-SEM (x-axis) and amplitude (bubble size) from the Lomb-Scargle analysis for data from Figs. 2, 3 and 4. (**G**) Comparison of baseline CDI (y-axis) with Tau-SEM (x-axis) and amplitude (bubble size) from the Chi-square analysis for data from Figs. 2, 3 and 4. (**H**) Comparison of baseline CDI (y-axis) with Tau-SEM (x-axis) and amplitude (bubble size) from the Wavelet analysis for data from Figs. 2, 3 and 4. R^2^ values are plotted on graphs (**E**–**H**) where the analysis was relevant.

This study explores the relationship between environmental disruption and circadian influence on locomotor behavior. A summary of the main environments studied, circadian behavior, and CDI scores are compared in Fig. 5A,B. The CDI tool we devised reliably and simply described the duration of locomotion and enhances the description of how different types of environmental light–dark conditions impact the circadian period and rhythmicity in mice. Correlating CDI to circadian periodicity (tau) as measured by Lomb-Scargle periodogram (Fig. 5C), chi-square periodogram (Fig. 5D), and wavelet analysis (Fig. 5E, R^2^ = 0.92) produced variable results for FDN-G compared to 16:8 LD and FDN-S conditions. However, the increased variability in circadian periodicity (tau) was strongly correlated with high CDI values for both FDN-G to DD and FDN-G to LL exposed mice as measured by the Lomb-Scargle periodogram (Fig. 5F, R^2^ = 0.92), chi-square periodogram (Fig. 5G, R^2^ = 0.70), and wavelet analysis (Fig. 5H, R^2^ = 0.87). There was a weaker correlation between periodicity (Tau-SEM) and amplitude (Supplementary Fig. S6). These findings indicate that using CDI as a predictive tool works best with free-running periods (shortened or lengthened).

## Discussion

With frequent exposure to light at night, daytime napping, and shift-work schedules, it is often difficult to maintain circadian photoentrainment. Our study demonstrates how light and dark exposure can impact three levels of circadian rhythm maintenance: (1) circadian photoentrainment, the most ideal for physiological homeostasis^4^, (2) circadian period lengthening, a disruptive deviation from circadian entrainment but still a positive indicator of a functional clock that is maintained under a number of disruptive T-cycles^28–32^, and (3) absent or suppressed circadian rhythmicity, the most disruptive state of the circadian clock that is usually associated with SCN (suprachiasmatic nucleus) ablation or mice with mutant clock gene components such as BMAL1^41–43^). Circadian arrhythmicity is the lack of a circadian rhythm. What this means numerically is less defined by chronobiology literature. We suggest that a combination of the following factors should be considered in determining locomotor circadian arrhythmicity: low amplitude of tau, highly variable tau (SEM used in this study), and a high CDI value. Although there was some disagreement between Lomb-Scargle, chi-square, and wavelet analyses, we demonstrated the three-fold change to amplitude (decreased), Tau-SEM (increased), and CDI (increased) for FDN-G to DD (Fig. 2, Days 1–5) and FDN-G-LL (Fig. 3) experimental groups. Overall, the phenotype for lack of rhythmicity for FDN-G mice was stronger in LL than in DD (Days 1–5). Unlike clock mutants, the effects of FDN-G were not permanent or long-term, as the circadian rhythm was recovered by 1-week of exposure to 12:12 LD and spontaneously after 5 days in DD. FDN-S mice recovered intrinsically-driven circadian rhythms during DD in phase with their lengthened rhythms during FDN-S, while FDN-G mice had a 5-day delay before recovery of a stable circadian rhythm, which demonstrates that the FDN-G environment disrupted behavioral circadian rhythms (Fig. 2). A similar delay in rhythm recovery in DD following a bifurcated circadian rhythm was also observed in hamsters in studies by Gorman and Elliot in 2003 and 2004 but contrasts with our study (FDN-G to DD, days 1–5) since the bifurcated rhythm was maintained upon initial DD exposure^44, 45^. Therefore, we propose that the FDN-G cycle could be used as a model for the temporary suppression of circadian behavioral rhythms in mice.

There have been numerous studies on disrupting circadian photoentrainment with light, however, inducing circadian arrhythmicity in mice using changes to the 24 h solar day has proven more elusive, although one study used extreme exposure to constant light (130 days) to eventually induce circadian arrhythmicity^10, 12, 16, 46^. There have also been studies in hamsters and chipmunks that demonstrate induction of circadian arrhythmicity using an approach seeded in phase response curve (PRC) theory, but these studies report unreliable induction of circadian arrhythmicity, especially in mice, and have not demonstrated a reversal approach^19–21^. Intriguingly, a study in hamsters shows arrhythmic animals with a strong light response in the SCN using *c-fos* gene expression studies, but a lack of a similar induction in the SCN of free-running animals indicating two separate responses of a non-entrained circadian clock^20^. These findings in hamsters suggest a strong possibility that the molecular responses responsible for differing phenotypes of the FDN-S and FDN-G mice would also vary. Future studies could be conducted to determine the validity of this hypothesis.

We are interested in light–dark disruptions that are socially relevant to the 24 h solar day. Some studies used non-24-h T-cycles with two separate light–dark periods (LDLD cycles), referred to as bifurcation, which disrupts circadian photoentrainment and results in the alignment of activity rhythms to the two periods of darkness and does not induce circadian arrhythmicity when exposed to constant dark conditions^12, 15, 16^. Although the achievement of bifurcation is regarded as advantageous for the adaptability of activity rhythms to extreme changes to the 24 h solar cycle (jet lag, shiftwork, space travel), considering bifurcation as a therapeutic option for daily adaptability requires the context of the solar day^47, 48^. Additionally, authors studying bifurcation including Gorman and Elliot, reported that sudden or gradual exposure to T18 or T30 yielded no difference in the ability of animals to achieve bifurcated rhythms^11, 12, 15, 16, 47^. These studies on bifurcation are different from the current study which shows how a gradual, not sudden, exposure to 4 LD cycles within 24 h (LDLDLDLD: quadfurcation), leads to circadian arrhythmicity of locomotion. A possible explanation for this phenomenon is that while the SCN can dissociate into two rhythms simultaneously, dissociation into four rhythms may be too strenuous to simultaneously maintain: the potential trigger for a transition to arrhythmicity^17, 18^. Furthermore, the sudden exposure to FDN over gradual exposure may favor free-running period induction as a protective measure to maintain some normalcy in the circadian system instead of an instant conversion to an arrhythmic output in the presence of light–dark environmental perturbations. Taken together with studies on LD bifurcation in hamsters, we uncover a threshold for disruptive LD fragmentation, where gradual LD quadfurcation (FDN-G) leads to behavioral circadian arrhythmicity in mice.

The timing of exposure to light and dark strongly influences the circadian clock and behavior^3, 49^. In our fragmented day-night paradigm, FDN-G mice aligned their activity with the dark fragments and avoided the light exquisitely, similar to the LDLD bifurcation paradigm, but in contrast, lost entrainment upon immediate exposure to constant dark and light conditions^11, 12, 15, 47^. Additionally, FDN-G mice instantly regained circadian rhythmicity following FDN-G upon exposure to the 1-week 12:12 LD cycle. FDN-G alignment with the dark fragments was not a circadian response, but likely another mechanism, since a similar finding was observed in BMAL1 and Per2/Cry1 mutants, which show similar alignment to LD cycles but suppressed rhythmicity in DD and LL^41, 50, 51^. This means that light is likely impacting behavior independently of a functional biological clock and has previously been described as an acute effect of light on behavior that overrides the influence of the circadian clock, known as negative masking^52, 53^. Therefore, the typical confinement of activity to the dark portion of the day in the FDN-G mice may not be completely under the control of the circadian clock, but rather acute light response drivers.

Researchers often employ wheel-running activity and the ClockLab software to record and analyze circadian rhythms in mice^22, 54–57^. Using data exported from ClockLab to increase the analytic resolution of our study, we examined multiple facets of circadian locomotion rhythms such as periodicity, amplitude, consistency of activity levels between animals, and the distribution of activity across the day. While there was a consistently suppressed amplitude measurement by the Lomb-Scargle, Chi-square, and wavelet analyses for the FDN-G, FDN-G to DD, and FDN-D to LL mice, periodicity (circadian tau) was highly variable. However, there was consistency in the high tau variability as measured by the SEM (Tau-SEM) for the FDN-G, FDN-G to DD, and FDN-D to LL, which enhances the conclusion that behavioral circadian rhythmicity was severely dampened in these mice. While there are many well-characterized approaches to measuring the rhythmicity of circadian behavior, measuring the duration of each phase of rhythmic behavior in a physiologically relevant manner is not well-studied^40^. The rationale for studying the active phase of circadian behavior using our CDI tool is important to describe the homeostatic drive and range of behavior, which is observed in many other biological processes such as body temperature and heart rate. We found a strong correlation between CDI and periodicity variability (Tau-SEM) with all three measures (Lomb-Scargle, Chi-square, and wavelet analyses), specifically for free-running conditions (Fig. 5). This validates the use of CDI as a potential predictive tool for variability in tau, which is important to determine confidence in the presence, or absence, of rhythmic behavior, such as locomotion. The CDI could be applied to a range of circadian-driven behaviors to determine the quantifiable fraction of the day different circadian behavior typically occupy, which will make the assessment of deviating behaviors easier to understand.

## Conclusion

Adaptation to a disruptive LD environment by confining activity to the available dark period may seem like a positive behavior for mice. However, here we show that is completely the opposite, as gradual adaptation and confinement to the four periods of darkness in the FDN-G paradigm leads to loss of behavioral circadian rhythmicity. The state of the circadian clock, as it relates to the external environment, is central to maintaining the synchronous internal balance necessary to support homeostatic mechanisms of a broad range of biological functions. Future studies would be necessary to determine the molecular state of the clock under FDN-G. This study highlights the importance of not only being aware of the presence of a disruptive light–dark environment but also whether the exposure was sudden or gradual. This study is relevant to designing environments that support daily rhythmic coordination in our bodies as well as recuperative health for the ill who are often exposed to irregular light schedules in hospitals and care facilities.

## Methods

### Mice

Age-matched C57BL/6NCrl males between 5 and 7 months old, were used for our experiments. Male mice were used in this study due to findings of increased variability in circadian-driven behaviors such as locomotion due to hormone variability associated with the 4–5 estrous cycle in female mice^2, 58^. For each figure, 3–4 sets of age-matched siblings (within 1 month of age of each other) were divided evenly among experimental groups with at least 2 siblings in each group. Future studies that can account for estrous-related variability will be considered in gendered circadian studies. Mice were individually housed in cages sized 6 × 9 × 18 inches with polycarbonate (transparent) material with food and water available ad libitum and treated in accordance with NIH, ARRIVE, and IACUC guidelines and used protocols approved by the Oakwood University Animal Care and Use Committee (OUACUC). Six mice were used per experimental group. Cage changes were done at the end of each experiment (most experiments were 1–2 weeks and did not require a cage change mid-experiment). For the FDN-G condition which was the longest (4 + weeks), the cages were quickly and carefully changed (30-s interaction with each mouse during the light phase) 1 day before switching to the 3 h split FDN-G cycle.

### Light cycles

#### Consolidated night (12:12 LD) cycle

12:12 LD is one of the most used light cycles to maintain and breed mice^59^. It consists of 12 h of darkness and 12 h of light that repeats every 24 h. Mice were bred and maintained under 12:12 LD before experiments. The light intensity used in our experiments was ~ 500 lx of white light (similar to office lighting- 32-Watt 4 ft. Linear T8 ALTO Fluorescent Tube Light Bulb Cool White).

#### Consolidated night (16:8 LD) cycle

Mice were transferred to the 16:8 LD cycle at least 2 weeks before any experimental changes in the light–dark environment occurred.

#### Fragmented day-night-sudden (FDN-S) cycle

The fragmented day-night cycle used in this study consists of 4-alternating cycles of 2 h of darkness and 4 h of light each 24-h day (also called T6 cycle in^36^). Mice were transferred immediately from 16:8 LD to FDN to create the FDN-S condition.

#### Fragmented day-night-gradual (FDN-G) cycle

Gradual exposure to the FDN cycle started with fragmenting the 8-h night into 2-h fragments, separated by 1 h of light for 1 week. Each subsequent week, the amount of light between fragments was increased by an hour to 2 h, 3 h, and eventually, the 4-h light exposure experience by the traditional FDN-S cycle explained above. Mice were maintained for 7–10 days in FDN-G before concurrent exposure to LL, DD, or LD paradigms (Figs. 2, 3, 4) (experimental details are outlined in Supplementary Tables S1–S4).

#### Constant darkness

Careful attention to prevent light pollution of any kind was taken during constant dark experimentation with the use of two layers of complete darkness: (1) the sealed (but still maintained airflow) dark experimentation cabinet, and (2) the locked, dark, and undisturbed room in which the cabinets are located.

#### The transition between light environments

Transitions were made as an extension of the previous light or dark cycle as appropriate for the environment being transitioned into. Light or dark was not introduced at times not indicated by the preceding or proceeding cycle in the gray-shaded areas on our actograms. For instance, FDN-S and FDN-G were transitioned to DD as an extension of the first dark period of the 4 fragments, while LL exposure was an extension of the fourth light period for the day before the first full day in LL.

### Circadian assessment of locomotion using wheel-running activity

Mice (male) were placed in cages with a 6-inch Actimetrics Wireless Low-profile Running Wheel. Analyses and monitoring of wheel-running activity were conducted with ClockLab (Actimetrics). Analyses include total activity, period length, and activity distribution across the light and dark periods of the day. Raw data for each animal were exported for statistical analysis. All the actograms are included in the Supplemental figures S1. The Lomb-Scargle periodogram was used to analyze amplitude and periodicity (tau). The activity onset for each free-running animal (LL and DD) was aligned to circadian time 12 using the Tau function (which matched the period calculation by the Lomb-Scargle periodogram) in the Actogram window of Clocklab, before exporting data for analysis and creating activity distribution graphs.

### Circadian duration index (CDI)

Circadian duration of activity across the 24-h day was determined by calculating the average wheel revolution in 30-min bins over 7 days for each mouse. The onset of activity (circadian time 12) was aligned with bin 1 (the first 30 min of analysis). If the circadian period was longer or shorter than 24 h, the tau was first adjusted to have the same onset time for activity each day before exporting daily averages. CDI = Time (in sums of 30 min bins) to accomplish 95% of daily locomotion/24. CDI scores of 0.33 and less are considered as strong indicators of consolidated locomotor behavior. CDI scores of 0.34–0.66 are considered moderate indicators of consolidated locomotor behavior. CDI scores of 0.67 or more are considered weak-absent indicators of consolidated locomotor behavior.

### Statistical analyses

Statistical analyses were conducted with Prism GraphPad, version 9.4.1. Six mice were used for each experiment; mice were not reused in different experiments. Where a comparison of three conditions was conducted, the one-way-ANOVA analysis was performed with a Tukey’s multiple comparisons post-test, because each data set was compared to the other two data sets. One-way ANOVA with repeated measures was used to assess supplementary Fig. S2. The alpha for all tests is 0.05. The distribution of data sets exhibited a Gaussian distribution of normality. The error bars represent the standard error of the mean. Descriptive statistics including the range, p-values, SEM, and F-values are included in each figure legend.

### Ethics declarations

This study aligns with the ARRIVE guidelines, uses enough mice for experiments, avoids invasive methods, and uses ethical practices.

### Supplementary Information

Supplementary Information.

## Data Availability

All data are available on reasonable request, and directed to the corresponding author, Melissa E S Richardson (mrichardson@oakwood.edu).

## References

[1] Blume C, Garbazza C, Spitschan M (2019). Effects of light on human circadian rhythms, sleep and mood. Somnologie..

[2] Jud C, Schmutz I, Hampp G, Oster H, Albrecht U (2005). A guideline for analyzing circadian wheel-running behavior in rodents under different lighting conditions. Biol. Proced. Online..

[3] Foster RG, Hughes S, Peirson SN (2020). Circadian photoentrainment in mice and humans. Biology..

[4] Bruce VG (1960). Environmental entrainment of circadian rhythms. Cold Spring Harb. Symp. Quant. Biol..

[5] Heyde I, Oster H (2019). Differentiating external zeitgeber impact on peripheral circadian clock resetting. Sci. Rep.

[6] Hasan S, Foster RG, Vyazovskiy VV, Peirson SN (2018). Effects of circadian misalignment on sleep in mice. Sci. Rep..

[7] Alves-Simoes M, Coleman G, Canal MM (2016). Effects of type of light on mouse circadian behaviour and stress levels. Lab. Anim..

[8] Schilperoort M (2020). Disruption of circadian rhythm by alternating light-dark cycles aggravates atherosclerosis development in APOE* 3-Leiden CETP mice. J. Pineal Res..

[9] Ameen RW, Warshawski A, Fu L, Antle MC (2022). Early life circadian rhythm disruption in mice alters brain and behavior in adulthood. Sci. Rep..

[10] Gorman MR (2001). Exotic photoperiods induce and entrain split circadian activity rhythms in hamsters. J. Comp. Physiol. A..

[11] Rosenthal SL, Vakili MM, Evans JA, Elliott JA, Gorman MR (2005). Influence of photoperiod and running wheel access on the entrainment of split circadian rhythms in hamsters. BMC Neurosci..

[12] Sun J, Joye DA, Farkas AH, Gorman MR (2019). Photoperiodic requirements for induction and maintenance of rhythm bifurcation and extraordinary entrainment in male mice. Clocks Sleep..

[13] Vilaplana J, Cambras T, Campuzano A, Díez-Noguera A (1997). Simultaneous manifestation of free-running and entrained rhythms in the rat motor activity explained by a multioscillatory system. Chronobiol. Int..

[14] Campuzano A, Vilaplana J, Cambras T, Díez-Noguera A (1998). Dissociation of the rat motor activity rhythm under T cycles shorter than 24 h. Physiol. Behav..

[15] Harrison E (2016). Extraordinary behavioral entrainment following circadian rhythm bifurcation in mice. Sci. Rep..

[16] Gorman MR, Elliott JA (2019). Focus: Clocks and cycles: Exceptional entrainment of circadian activity rhythms with manipulations of rhythm waveform in male Syrian hamsters. YJBM..

[17] de la Iglesia, H. O., Carpino, M. J., & Schwartz, W. J. Antiphase oscillation of the left and right suprachiasmatic nuclei. *Science*. **290**(5492), 799–801 (2000). 10.1126/science.290.5492.799 11052942

[18] Ono D, Honma S, Nakajima Y, Kuroda S, Enoki R, Honma KI (2017). Dissociation of Per1 and Bmal1 circadian rhythms in the suprachiasmatic nucleus in parallel with behavioral outputs. PNAS.

[19] Ruby NF, Barakat MT, Heller HC (2004). Phenotypic differences in reentrainment behavior and sensitivity to nighttime light pulses in Siberian hamsters. J. Biol. Rhythms..

[20] Ruby NF (2021). Suppression of circadian timing and its impact on the hippocampus. Front. Neurosci..

[21] Honma S, Honma KI (1999). Light-induced uncoupling of multioscillatory circadian system in a diurnal rodent, Asian chipmunk. Am. J. Physiol. Regul. Integr. Comp. Physiol..

[22] Eckel-Mahan K, Sassone-Corsi P (2015). Phenotyping circadian rhythms in mice. Curr. Proc..

[23] Hughes ATL (2021). Timed daily exercise remodels circadian rhythms in mice. Commun. Biol..

[24] Aton SJ, Block GD, Tei H, Yamazaki S, Herzog ED (2004). Plasticity of circadian behavior and the suprachiasmatic nucleus following exposure to non-24-hour light cycles. J. Biol. Rhythms..

[25] Hughes AT (2015). Constant light enhances synchrony among circadian clock cells and promotes behavioral rhythms in VPAC2-signaling deficient mice. Sci. Rep..

[26] Menaker, M. Extraretinal light perception in the sparrow. I. Entrainment of the biological clock. *PNAS* **59**(2), 414–421 (1968). 10.1073/pnas.59.2.414 PMC224688 5238974

[27] Aschoff J (1979). Circadian rhythms: Influences of internal and external factors on the period measured in constant conditions. Z. Tierpsychol..

[28] Chen R, Seo DO, Bell E, Von Gall C, Lee C (2008). Strong resetting of the mammalian clock by constant light followed by constant darkness. J. Neurosci..

[29] Park N, Cheon S, Son GH, Cho S, Kim K (2012). Chronic circadian disturbance by a shortened light-dark cycle increases mortality. Neurobiol. Aging..

[30] Altimus CM (2008). Rods-cones and melanopsin detect light and dark to modulate sleep independent of image formation. PNAS.

[31] LeGates TA (2012). Aberrant light directly impairs mood and learning through melanopsin-expressing neurons. Nature.

[32] Ono D, Honma S, Honma KI (2013). Postnatal constant light compensates Cryptochrome1 and 2 double deficiency for disruption of circadian behavioral rhythms in mice under constant dark. PLoS ONE.

[33] Ohta H, Mitchell A, McMahon D (2006). Constant light disrupts the developing mouse biological clock. Pediatr. Res..

[34] Oliver PL (2012). Disrupted circadian rhythms in a mouse model of schizophrenia. Curr. Biol..

[35] Tapia-Osorio A, Salgado-Delgado R, Angeles-Castellanos M, Escobar C (2013). Disruption of circadian rhythms due to chronic constant light leads to depressive and anxiety-like behaviors in the rat. Behav. Brain Res..

[36] Richardson, M. E., Brown, D., Honore, D., & Labossiere, A. Fragmented day-night cycle induces period lengthening, lowered anxiety, and anhedonia in male mice. *Behav. Brain Res.* **413**, 113453. 10.1016/j.bbr.2021.113453 (2021). 10.1016/j.bbr.2021.113453 34252503

[37] Richardson, M. E., Boutrin, M. C., Chunn, S., & Hall, M. Fragmented day-night cycle induces reduced light avoidance, excessive weight gain during early development, and binge-like eating during adulthood in mice. *Physiol. Behav.* 113851. 10.1016/j.physbeh.2022.113851 (2022). 10.1016/j.physbeh.2022.113851 35609722

[38] Ruf T (1999). The lomb-scargle periodogram in biological rhythm research: Analysis of incomplete and unequally spaced time-series. Biol. Rhythm Res..

[39] Tackenberg MC, Hughey JJ (2021). The risks of using the chi-square periodogram to estimate the period of biological rhythms. PLoS Comput. Biol..

[40] Leise TL (2017). Analysis of nonstationary time series for biological rhythms research. J. Biol. Rhythms..

[41] Izumo, M., *et al.* Differential effects of light and feeding on circadian organization of peripheral clocks in a forebrain Bmal1 mutant. *Elife*. **3**, 04617. 10.7554/eLife.04617 (2014). 10.7554/eLife.04617 PMC4298698 25525750

[42] Abe YO (2022). Rhythmic transcription of Bmal1 stabilizes the circadian timekeeping system in mammals. Nat. Commun..

[43] Tso CF (2017). Astrocytes regulate daily rhythms in the suprachiasmatic nucleus and behavior. Curr. Biol..

[44] Gorman MR, Elliott JA (2003). Entrainment of 2 subjective nights by daily light:dark:light:dark cycles in 3 rodent species. J. Biol. Rhythms..

[45] Gorman MR, Elliott JA (2004). Dim nocturnal illumination alters coupling of circadian pacemakers in Siberian hamsters, Phodopus sungorus. J. Comp. Physiol..

[46] Ohta H, Yamazaki S, McMahon DG (2005). Constant light desynchronizes mammalian clock neurons. Nat. Neurosci..

[47] Walbeek TJ, Harrison EM, Soler RR, Gorman MR (2019). Enhanced circadian entrainment in mice and its utility under human shiftwork schedules. Clocks Sleep..

[48] Noguchi T (2020). Circadian rhythm bifurcation induces flexible phase resetting by reducing circadian amplitude. Eur. J. Neurosci..

[49] Li Y, Androulakis IP (2021). Light entrainment of the SCN circadian clock and implications for personalized alterations of corticosterone rhythms in shift work and jet lag. Sci. Rep..

[50] Abraham D (2006). Restoration of circadian rhythmicity in circadian clock-deficient mice in constant light. J. Biol. Rhythms..

[51] Bittman EL (2021). Entrainment is NOT synchronization: An important distinction and its implications. J. Biol. Rhythms..

[52] Mrosovsky N (1999). Masking: History, definitions, and measurement. Chronobiol. Int..

[53] Altimus, C. M., LeGates, T. A., & Hattar, S. Circadian and Light Modulation of Behavior. in *Mood and Anxiety Related Phenotypes in Mice,* pp. 47–65 (Humana Press, Totowa, NJ, 2009).

[54] Siepka SM, Takahashi JS (2005). Methods to record circadian rhythm wheel running activity in mice. Meth. Enzymol..

[55] Brown LA, Fisk AS, Pothecary CA, Peirson SN (2019). Telling the time with a broken clock: Quantifying circadian disruption in animal models. Biology..

[56] Verwey, M., Robinson, B., & Amir, S. Recording and analysis of circadian rhythms in running-wheel activity in rodents. *JoVE*. **71**, 50186 (2013) 10.3791/50186 PMC3582575 23380887

[57] Refinetti R, Cornélissen G, Halberg F (2007). Procedures for numerical analysis of circadian rhythms. Biol. Rhythm Res..

[58] Datta S, Samanta D, Sinha P, Chakrabarti N (2016). Gender features and estrous cycle variations of nocturnal behavior of mice after a single exposure to light at night. Physiol. Behav..

[59] Jennings M (1998). Refining rodent husbandry: The mouse: Report of the rodent refinement working party. Lab. Anim..

