# Supplementary material for: Reversible suppression of circadian-driven locomotor rhythms in mice using a gradual fragmentation of the day-night cycle
Source: Sci Rep. 2023 Sep 2;13:14423. doi: 10.1038/s41598-023-41029-0 (PMC10475134; doi:10.1038/s41598-023-41029-0)
Supplement: Supplementary file 1 — Supplementary Information. [file 41598_2023_41029_MOESM1_ESM.docx]

Supplementary Table 1

**Figure 1a- Concurrent experimental design**

| Light cycle #1 | | Light cycle #2 | | Light cycle #3 | | Light cycle #4 | | Light cycle #5 | |
| --- | --- | --- | --- | --- | --- | --- | --- | --- | --- |
| Exp. | Days | Exp. | Days | Exp. | Days | Exp. | Days | Exp. | Days |
| **16:8 LD** | 14 |  |  |  |  |  |  |  |  |
| 16:8 LD | 14 | **FDN-S** | **10** |  |  |  |  |  |  |
| 16:8 LD | 14 | FDN-G 1hr split | 7 | FDN-G 2hr split | 7 | FDN-G 3hr split | 7 | **FDN-G 4hr split** | **14** |
| **Analysis on the *last* 7 days only** | | | | | | | | | |
| **Analysis on the first 7 days only** | | | | | | | | | |

**Concurrent experiments**

Each row represents 1 group with 6 mice. Light cycle #1 is when all experiments began concurrently. Due to the varying time requirements for each experiment, there was a staggered termination for each experimental group. Gray boxes indicate times of no experiment for each group.

Supplementary Figure S1
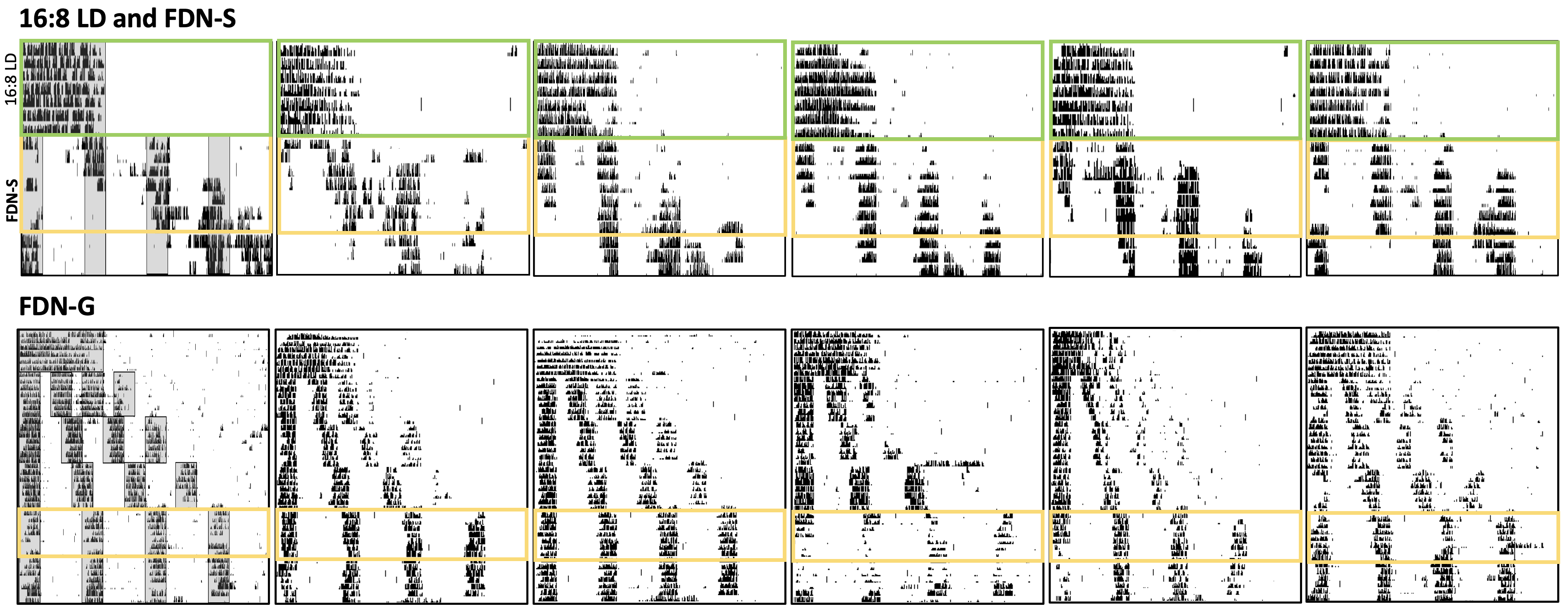


**All actograms for Figure 1 analysis.**

Supplementary Figure S2


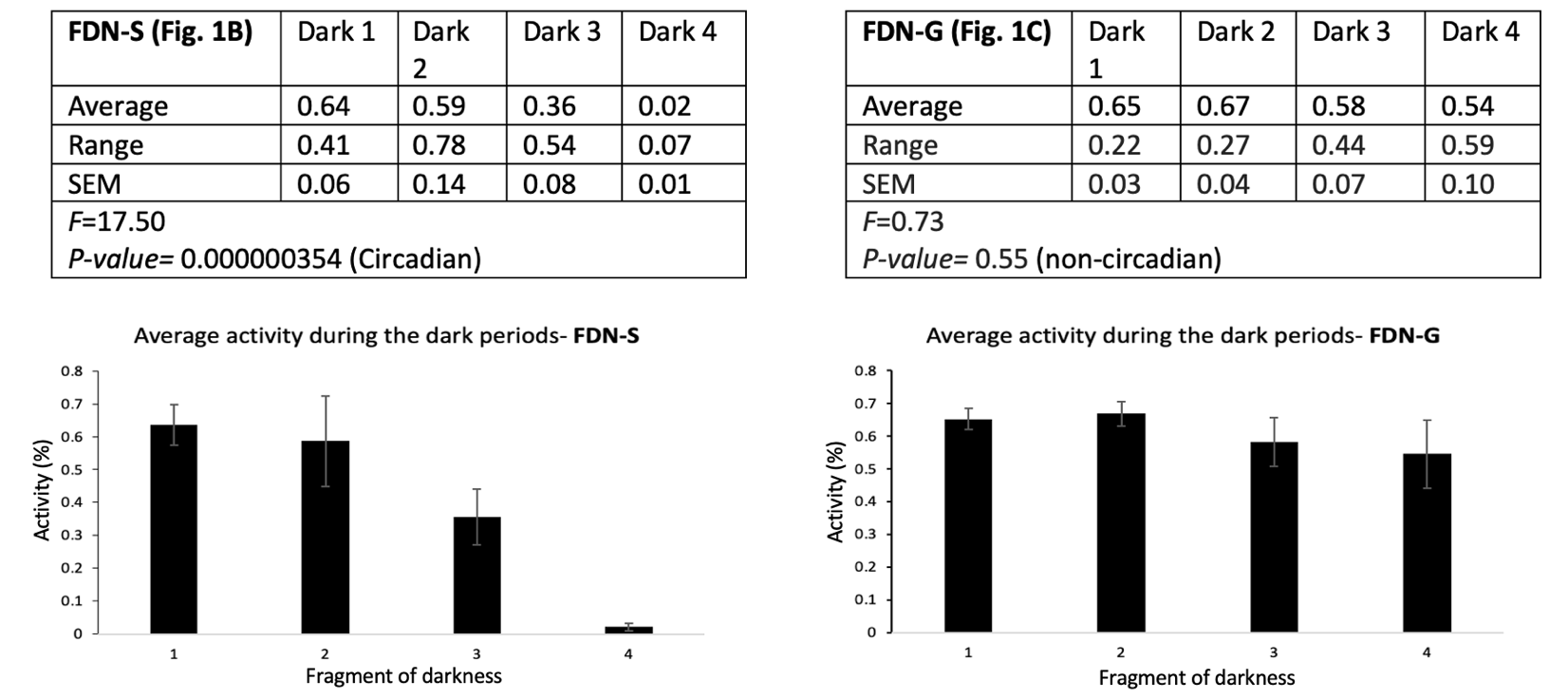


**Comparison of activity levels during the dark periods of FDN-S vs FDN-G.** Statistical comparison was made using one-way ANOVA with repeated measures. *Supplementary to Figure 1.*

Supplementary Figure S3


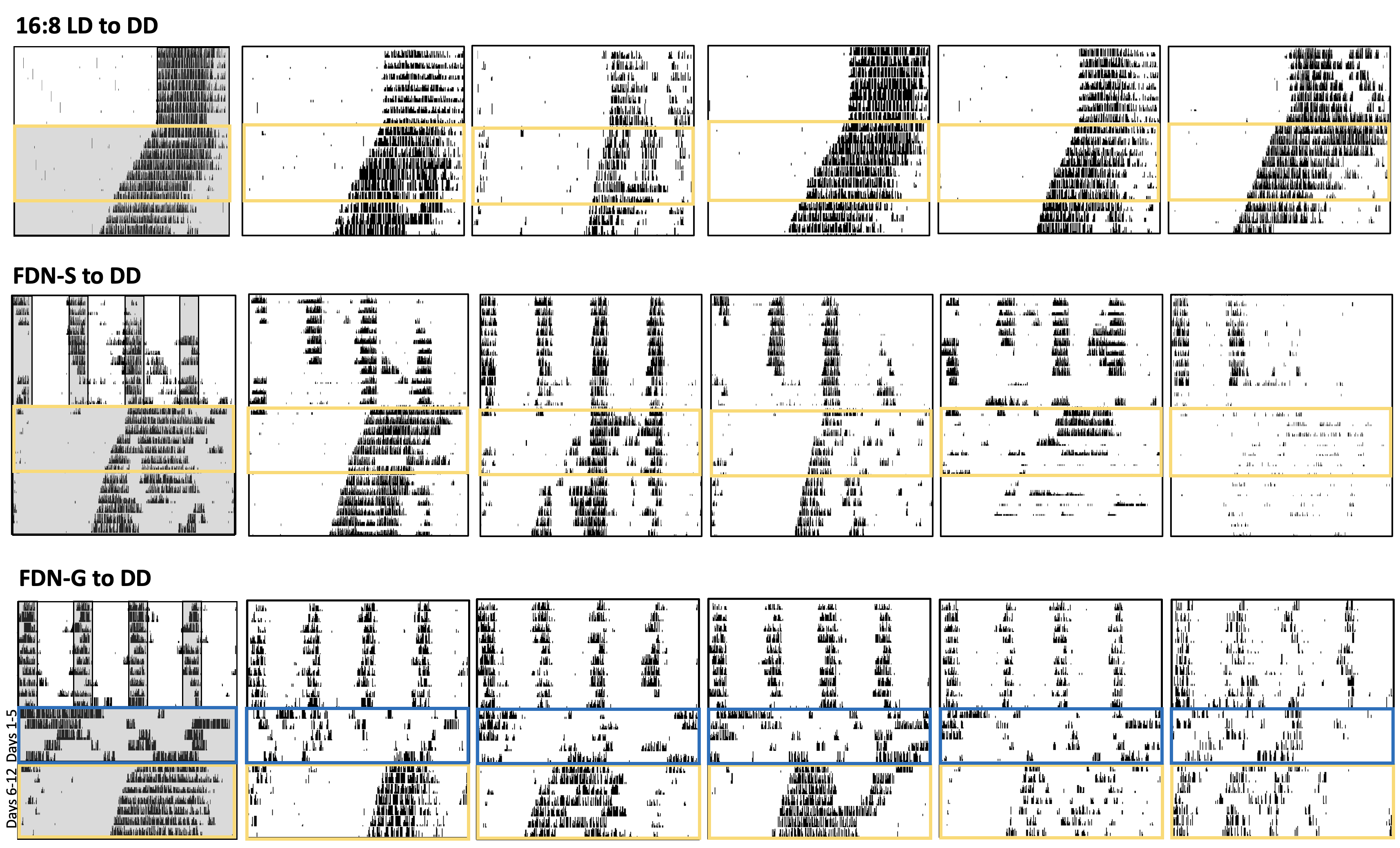


**All actograms for Figure 2 analysis.**

Supplementary Table 2

**Figure 2a- Concurrent experimental design**

| Light cycle #1 | | Light cycle #2 | | Light cycle #3 | | Light cycle #4 | | Light cycle #5 | | Light cycle #6 | |
| --- | --- | --- | --- | --- | --- | --- | --- | --- | --- | --- | --- |
| Exp. | Days | Exp. | Days | Exp. | Days | Exp. | Days | Exp. | Days | Exp. | Days |
|  |  |  |  |  |  |  |  | 16:8 LD | 14 | **DD** | **10** |
|  |  |  |  |  |  | 16:8 LD | 14 | FDN-S | 10 | **DD** | **13** |
| 16:8 LD | 14 | FDN-G 1hr split | 7 | FDN-G 2hr split | 7 | FDN-G 3hr split | 7 | FDN-G 4hr split | 10 | **DD** | **12** |
| **Analysis on the first 7 days only** | | | | | | | | | | | |

**Concurrent experiments**

Each row represents 1 group with 6 mice. Light cycle #6 is when all experimental groups were exposed to DD. Due to the varying time requirements for each experiment, there was a staggered start for each experimental group. Gray boxes indicate times of no experiment for each group. Mice were allowed to stay in the Light cycle #6 (DD) for a variable number of days after the initial 7 days used for analysis for observational purposes.

Supplementary Figure S4


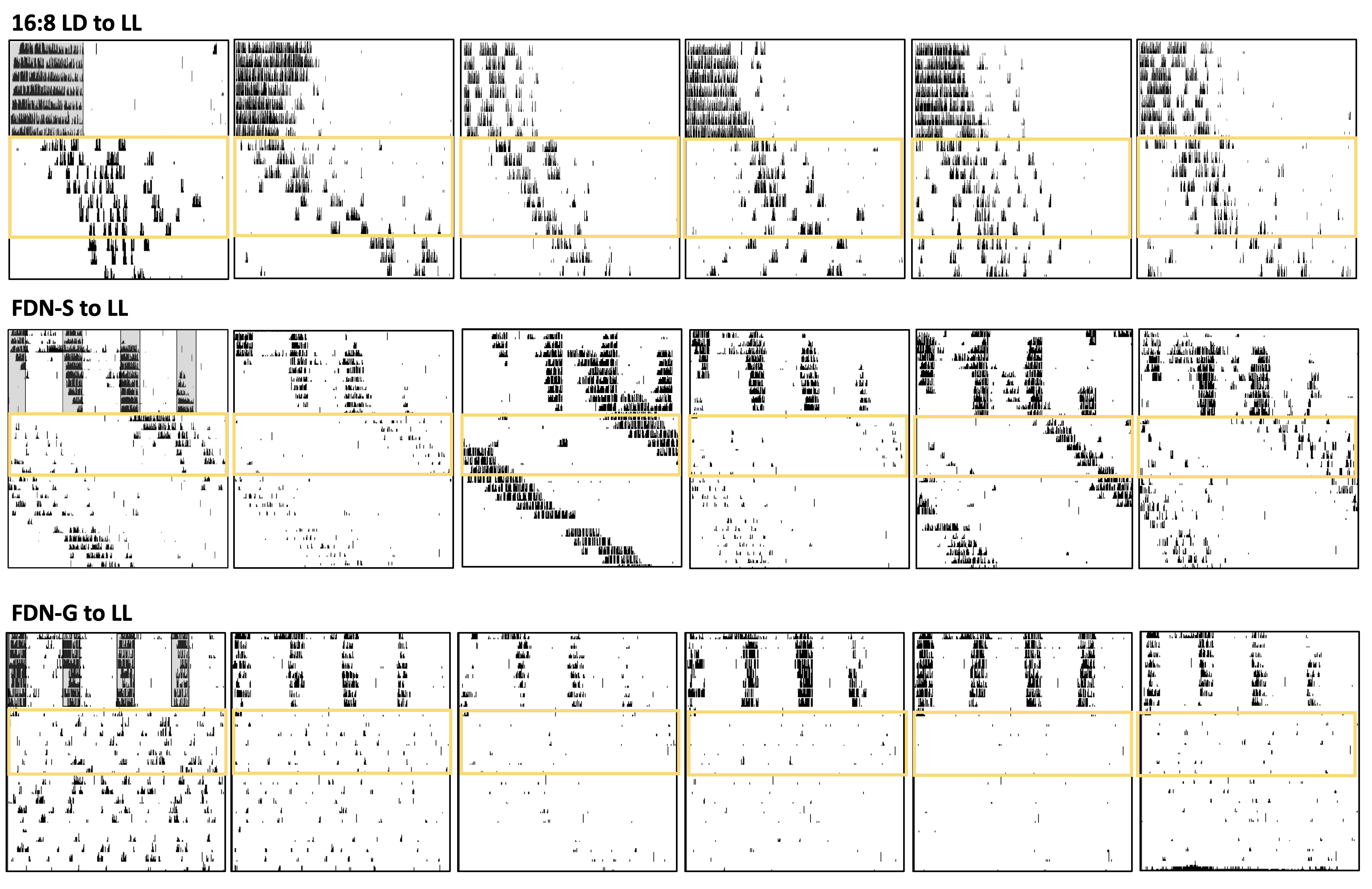


**All actograms for Figure 3 analysis.**

Supplementary Table 3

**Figure 3a- Concurrent experimental design**

| Light cycle #1 | | Light cycle #2 | | Light cycle #3 | | Light cycle #4 | | Light cycle #5 | | Light cycle #6 | |
| --- | --- | --- | --- | --- | --- | --- | --- | --- | --- | --- | --- |
| Exp. | Days | Exp. | Days | Exp. | Days | Exp. | Days | Exp. | Days | Exp. | Days |
|  |  |  |  |  |  |  |  | 16:8 LD | 14 | **LL** | **10** |
|  |  |  |  |  |  | 16:8 LD | 14 | FDN-S | 10 | **LL** | **18** |
| 16:8 LD | 14 | FDN-G 1hr split | 7 | FDN-G 2hr split | 7 | FDN-G 3hr split | 7 | FDN-G 4hr split | 10 | **LL** | **17** |
| **Analysis on the first 7 days only** | | | | | | | | | | | |

**Concurrent experiments**

Each row represents 1 group with 6 mice. Light cycle #6 is when all experimental groups were exposed to LL. Due to the varying time requirements for each experiment, there was a staggered start for each experimental group. Gray boxes indicate times of no experiment for each group. Mice were allowed to stay in the Light cycle #6 (LL) for a variable number of days after the initial 7 days used for analysis for observational purposes.

Supplementary Figure S5


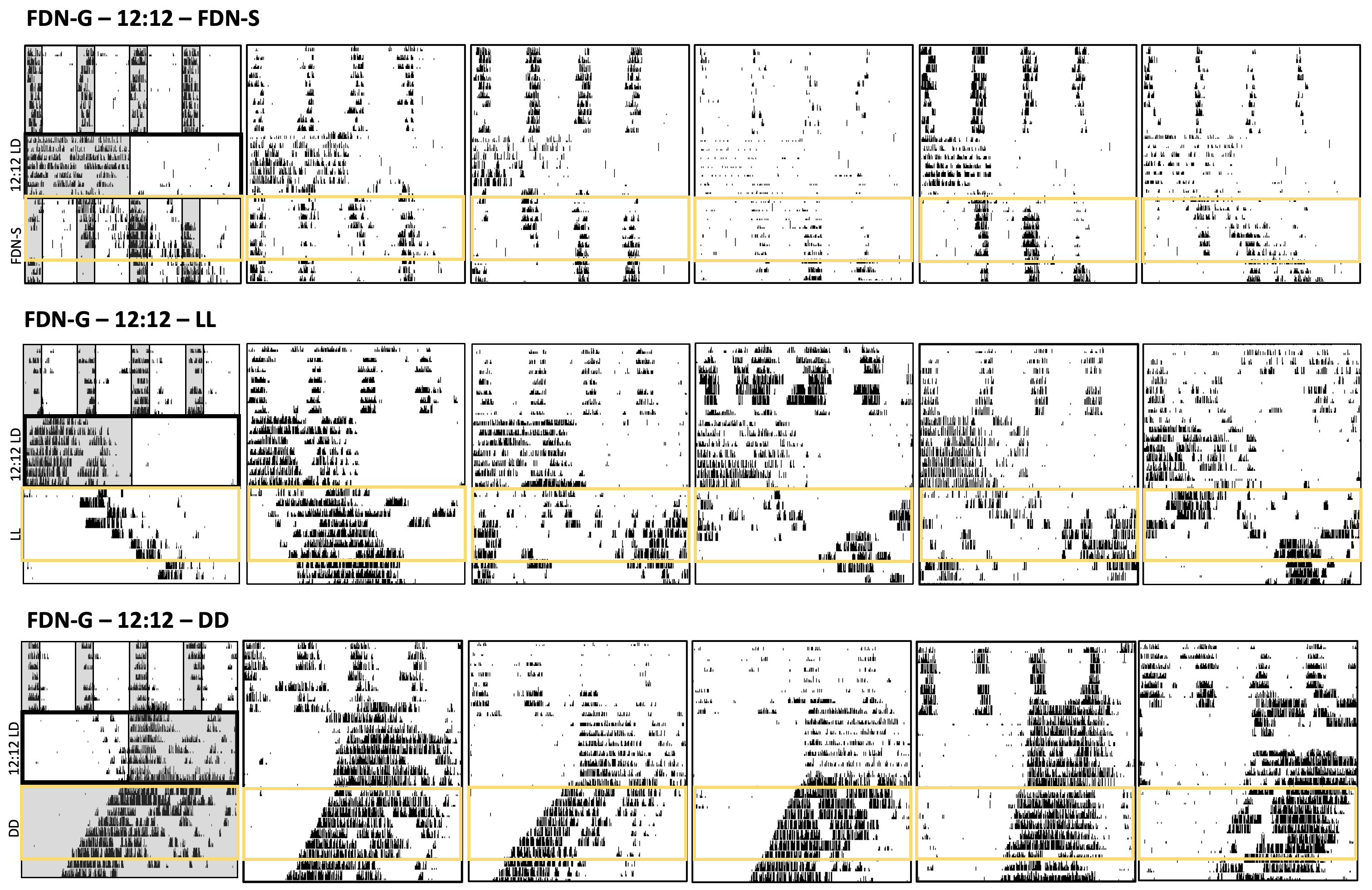


**All actograms for Figure 4 analysis.**

Supplementary Table 4

**Figure 4a- Concurrent experimental design**

| Light cycle #1 | | Light cycle #2 | | Light cycle #3 | | Light cycle #4 | | Light cycle #5 | | Light cycle #6 | | Light cycle #7 | |
| --- | --- | --- | --- | --- | --- | --- | --- | --- | --- | --- | --- | --- | --- |
| Exp. | Days | Exp. | Days | Exp. | Days | Exp. | Days | Exp. | Days | Exp. | Days | Exp. | Days |
| 16:8 LD | 14 | FDN-G 1hr split | 7 | FDN-G 2hr split | 7 | FDN-G 3hr split | 7 | FDN-G 4hr split | 10 | 12:12 LD | 7 | **FDN-S** | **9** |
| 16:8 LD | 14 | FDN-G 1hr split | 7 | FDN-G 2hr split | 7 | FDN-G 3hr split | 7 | FDN-G 4hr split | 10 | 12:12 LD | 7 | **DD** | **9** |
| 16:8 LD | 14 | FDN-G 1hr split | 7 | FDN-G 2hr split | 7 | FDN-G 3hr split | 7 | FDN-G 4hr split | 10 | 12:12 LD | 7 | **LL** | **9** |
| **Analysis on the first 7 days only** | | | | | | | | | | | | | |

**Concurrent experiments**

Each row represents 1 group with 6 mice. Light cycle #7 is when all experimental groups were exposed to FDN-S, DD, or LL. Mice were allowed to stay in Light cycle #7 for a variable number of days after the initial 7 days used for analysis for observational purposes.

Supplementary Figure S6


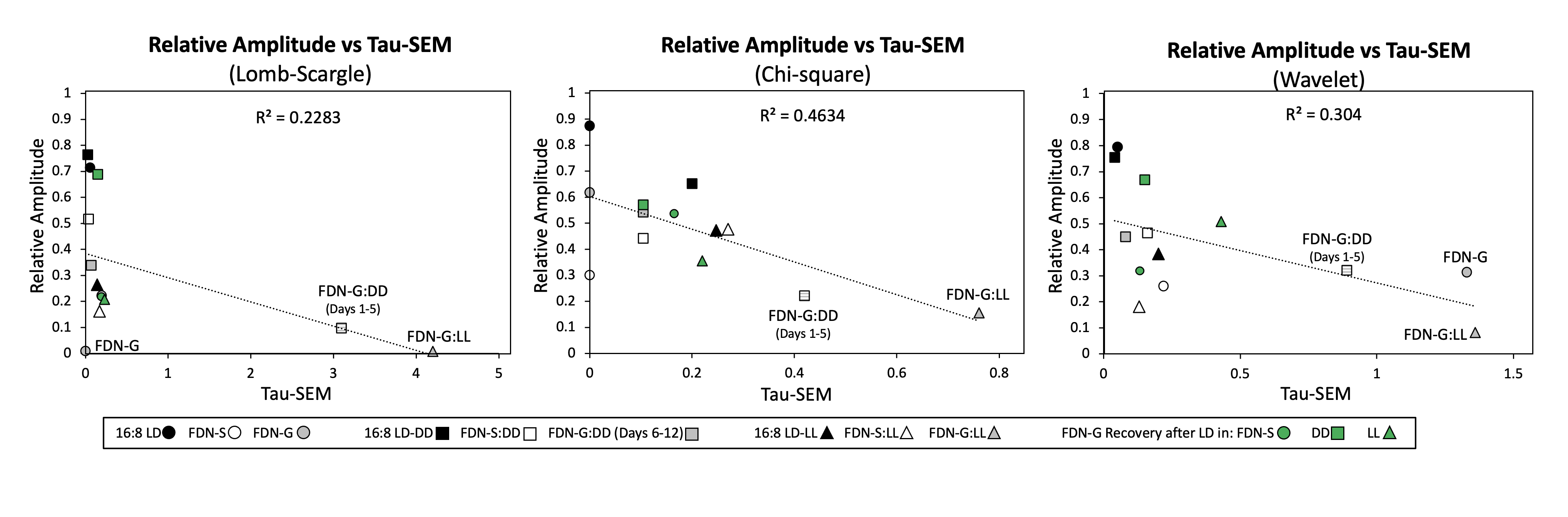


Comparison of Relative amplitude to Tau-SEM across all environments. Amplitude was normalized to the highest amplitude value for Lomb-Scargle, Chi-square, and Wavelet data sets. R^2^ values are plotted on graphs.
